# A self-guided and monitored digital problem-solving intervention for patients with symptoms of depression or anxiety on the waiting list for treatment in routine psychiatric care: feasibility study

**DOI:** 10.1192/bjo.2022.14

**Published:** 2022-02-08

**Authors:** Amira Hentati, Erik Forsell, Brjánn Ljótsson, Nils Lindefors, Martin Kraepelien

**Affiliations:** Centre for Psychiatry Research, Department of Clinical Neuroscience, Karolinska Institutet & Stockholm Health Care Services, Region Stockholm, Sweden; and Division of Psychology, Department of Clinical Neuroscience, Karolinska Institutet, Sweden; Centre for Psychiatry Research, Department of Clinical Neuroscience, Karolinska Institutet & Stockholm Health Care Services, Region Stockholm, Sweden; and Division of Psychology, Department of Clinical Neuroscience, Karolinska Institutet, Sweden; Division of Psychology, Department of Clinical Neuroscience, Karolinska Institutet & Stockholm Health Care Services, Region Stockholm, Sweden; Centre for Psychiatry Research, Department of Clinical Neuroscience, Karolinska Institutet & Stockholm Health Care Services, Region Stockholm, Sweden; and Division of Psychology, Department of Clinical Neuroscience, Karolinska Institutet, Sweden; Centre for Psychiatry Research, Department of Clinical Neuroscience, Karolinska Institutet & Stockholm Health Care Services, Region Stockholm, Sweden; and Division of Psychology, Department of Clinical Neuroscience, Karolinska Institutet, Sweden

**Keywords:** Anxiety disorders, cognitive–behavioural therapies, depressive disorders, digital intervention, routine psychiatry

## Abstract

**Background:**

There is often a waiting period for people who seek psychiatric treatment for depression or anxiety. As this delay risks worsening symptoms, an alternative could be to provide an intervention that requires minimal resources during the waiting period.

**Aims:**

The aim was to investigate if a digital problem-solving intervention delivered in a self-guided format with automated features is feasible to provide for patients on the waiting list in routine psychiatric care.

**Method:**

A total of 12 patients with symptoms of depression or anxiety on the waiting list for treatment in routine psychiatric care were given access to a self-guided and monitored digital problem-solving intervention over 4 weeks. Primary outcome measures were treatment credibility and usability. Secondary outcome measures were behavioural engagement, symptoms of depression and anxiety, and negative effects.

**Results:**

A majority of participants rated the intervention as both credible and usable. The intervention was used at least once by nine out of 12 individuals, with an average of 11 logins. The participants did, on average, initiate 2.8 problem-solving attempts and 10.1 solutions. A few participants reached a clinically relevant symptom improvement of depression and anxiety. No serious negative effects were reported.

**Conclusions:**

The credibility and usability of the intervention was perceived as good, and the behavioural engagement with the intervention was deemed sufficient compared with similar self-guided interventions. A self-guided and monitored digital problem-solving intervention may be a beneficial option for patients waiting for or receiving treatment in routine psychiatric care, and should be further evaluated.

## Access to treatment

A large number of patients suffer from depression and anxiety worldwide.^1^ The conditions cause extensive disability and societal costs.^2,3^ There are effective treatments for both depression and anxiety, and cognitive–behavioural therapy (CBT) is an evidence-based psychological treatment for both conditions when delivered face to face or in an internet-delivered format.^4^ However, access to psychological treatment is limited for a number of reasons, such as the limited number of therapists,^5^ resulting in some patients not accessing treatment or having to wait for access. Being on the waiting list for treatment may be associated with worsening of symptoms and more suffering,^6^ or at the very least a prolonged suffering and waste of time that could be spent starting to improve. A possible option could be to provide patients with an easily administered intervention that is scalable and requires minimal resources, while awaiting access to treatment. This could be considered part of a stepped-care model, where patients gain access to a self-care intervention early on in the care process.^7^

## Problem-solving therapy

Problem-solving therapy is a well-established and evidence-based intervention originally developed for depression, but also provided for patients with anxiety syndromes,^8–10^ and has shown to be effective in several meta-analyses.^11,12^ The intervention focuses on training adaptive problem-solving skills to cope more effectively with a range of difficult and stressful situations and problems,^12^ and has been delivered in a face-to-face format^11^ as well as a digital format.^9^ When delivered in a digital format, the intervention has been delivered both with therapist guidance and in a self-guided format.^9^

Although the problem-solving intervention has shown to be more effective in reducing symptoms of depression and anxiety when delivered in a therapist-guided format compared with a self-guided format,^9^ the intervention has not yet been assessed in a self-guided format with the addition of automated features and monitoring of patients’ symptoms. There are indications that such features could enhance the effects of self-guided interventions.^13,14^

A problem-solving intervention with online written therapist guidance has been tested on patients with symptoms of depression and anxiety on the waiting list for treatment at a Dutch mental health centre.^15^ In this study, around half of patients opted to receive the therapist-guided problem-solving intervention while waiting for treatment. In addition to this published study, a protocol of a guided digital intervention, including problem-solving components, for patients with major depression who were on the waiting list for treatment has also been published.^16^ Problem-solving has furthermore been shown to be an acceptable part of a video-based intervention provided for patients in a psychiatric waiting room area.^17^ Moreover, some design implications of a digital intervention supporting patients during pre-therapy waiting periods have been described in a conference presentation,^18^ but this intervention has not yet been empirically examined. It is, however, still unclear whether a digital problem-solving intervention provided in a self-guided format with automated features is feasible in a routine psychiatric care setting.

## Feasibility aspects

When delivering healthcare interventions in a digital format, treatment adherence has been shown to increase when patients are content with the intervention and delivery mode.^19^ Thus, when developing new digital healthcare interventions, it is important to involve patients and consider their perspectives on the intervention and treatment format.^20^

A factor strongly linked to treatment satisfaction and adherence is treatment credibility, referring to an individual's belief in an intervention.^21^ Perceiving an intervention to be credible has shown to be positively related to adherence and treatment outcome, and negatively related to dropping out of treatment.^21,22^ Other specific factors associated with increased adherence is if the intervention is perceived as helpful and relevant to the patient's problems.^19^

Since digital interventions can be efficient only if patients interact with them,^20^ a vital factor when delivering digital healthcare is the ease of use of a system, also known as usability.^23^ As with insufficient treatment credibility, inadequate usability has been linked to both non-usage and low adherence to treatment.^24,25^

Assesing the behavioural engagement with an intervention is a useful complement to the perceived satisfaction with an intervention and its format.^26^ Conventionally, behavioural engagement with digital interventions is measured by the completion of intervention-related content and/or exercises, as well as by the number of logins to the treatment platform.^27^ Furthermore, although the focus of a feasibility evaluation should be on whether the intended intervention and study procedure is feasible in a particular setting, rather than on treatment effectiveness, intended outcome measures should be included to enable the evaluation of the assessment procedures.^28^

## Aim

The aim of this study was to evaluate the feasibility of a self-guided and monitored digital problem-solving intervention for patients with symptoms of depression or anxiety who are awaiting treatment in routine psychiatric care in Sweden. Feasibility was to be measured by self-rated treatment credibility and usability, behavioural engagement and preliminary effects on symptoms of depression and anxiety.

## Method

### Setting and study design

This study investigated the feasibility of a self-guided and monitored digital problem-solving intervention provided to patients with symptoms of depression or anxiety who are between the assessment phase and the treatment phase within routine psychiatric care in Sweden.

The authors assert that all procedures contributing to this work comply with the ethical standards of the relevant national and institutional committees on human experimentation and with the Helsinki Declaration of 1975, as revised in 2008. All procedures involving human patients were approved by the Swedish National Ethical Review Board (identifier 2019-05911). This trial was pre-registered on ClinicalTrials.gov (identifier NCT04277793) on 20 February 2020.

### Participants and recruitment

The recruitment was conducted within a routine psychiatric care unit in Sweden during the spring and autumn of 2020. Participants were informed of the study through information from the healthcare personnel at the psychiatric care unit. During the recruitment period, restrictions relating to the COVID-19 pandemic were introduced into Swedish healthcare, resulting in fewer patient visits at the unit, and prolonging the planned recruitment period by around 4 months.

Inclusion criteria were as follows: age ≥18 years; on the waiting list for treatment within routine psychiatric care; and presence of clinically significant symptoms of depression or anxiety, as measured by scoring ≥5 points on the standardised and self-assessed questionnaires Patient Health Questionnaire-9 (PHQ-9)^29^ and/or Generalized Anxiety Disorder-7 (GAD-7).^30^ Exclusion criteria were as follows: insufficient knowledge of the Swedish language, lack of access to mobile or desktop device with an internet connection, lack of access to a telephone that could receive calls and text messages, severe suicidal ideation as measured by ≥4 points on the ninth item of Montgomery–Åsberg Depression Rating Scale – Self Assessment (MADRS-S)^31^ and psychiatric or somatic difficulties that should be prioritised or would constitute an obstacle for the intervention.

### Procedures

**Fig. 1:**
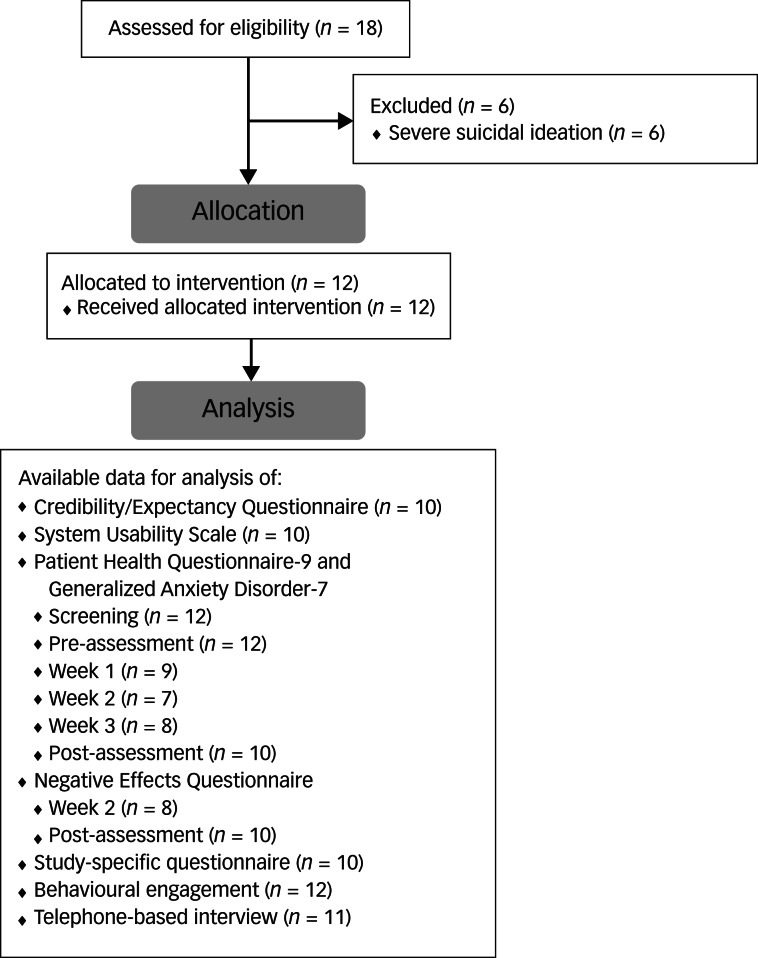
Flow chart.

Study registration was self-administered online on a secure platform. All individuals who registered to the study were contacted and informed of whether they qualified to participate. Those who were included received a brief introduction to the intervention by telephone. Written informed consent was obtained from all patients digitally. A total of 18 persons from the psychiatric care unit registered to the study. Six individuals were excluded from the study because they met the exclusion criterion concerning severe suicidal ideation. Thus, a total of 12 participants were included in the study and received access to a self-guided and monitored digital problem-solving intervention for a period of 4 weeks. See Figure 1 for the study flow chart.

### Intervention

The problem-solving intervention was hosted on a secure digital treatment platform and could be accessed via both computer and mobile devices connected to the internet. The content was based on an already existing digital problem-solving intervention used as a component in internet-based CBT in Swedish regular healthcare for individuals with major depression,^32^ and was adapted for self-guidance.

The intervention comprised psychoeducational texts, treatment rationale, examples of problems and suggestions of solutions, illustrative pictures, instructions and problem-solving exercises. All content was presented in Swedish, and the intervention consisted of about 4800 words. Reminders to remember to use the intervention were sent to all participants by mobile text messages during the access period. In addition, an automated e-mail was sent to all participants each week during the access period, containing a weekly suggestion related to the intervention.

**Fig. 2:**
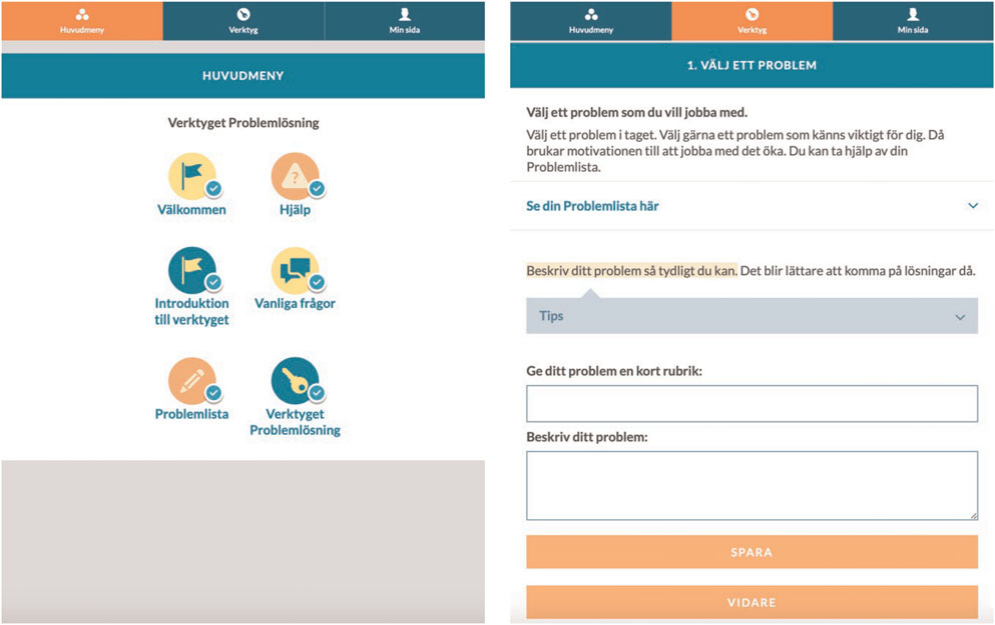
Main menu (left) and the first step of the problem-solving intervention (right), in Swedish.

The user interface of the intervention was developed in collaboration with experts on user experience design, to make the intervention simple and intuitive to use in a self-guided format. Several features were built into the intervention to facilitate self-guided usage. First, because of increased mobile device usage,^33^ the user interface was designed with the responsive design method mobile-first, meaning that the mobile version of the product was designed first, and was thereafter expanded to a desktop version. Second, the navigation menu comprised text accompanied by pictograms, to visually differentiate content. Third, content was divided into different sections, subsections, pages and expandable learn-more options for the purpose of limiting the amount of content displayed simultaneously. This made it possible to present the content in small chunks. Fourth, the intervention itself was presented in a stepwise fashion. Each step in the problem-solving intervention came with its own instructions, and every new step was depended on information that had been entered in previous steps. Finally, automatic pop-ups were built into the intervention to expose the participants to helpful questions related to the exercises, as well as encouragement to keep on working with the exercises. See Figure 2 for a visual presentation of parts of the problem-solving intervention.

### Measurements

When registering to the study, participants completed a digital assessment with questions regarding demographics, as well as inclusion and exclusion criteria. As a part of the assessment of eligibility, participants completed the standardised and self-assessed questionnaires PHQ-9^29^ and GAD-7,^30^ measuring symptoms of depression and anxiety, respectively, and the ninth item of MADRS-S,^31^ concerning suicidal ideation. The PHQ-9 ranges from 0 to 3 points per item and 0 to 27 points in total. The corresponding points for the GAD-7 are 0–3 points per item and 0–21 points in total. The higher score on the PHQ-9 and GAD-7, the more symptoms on the respective scale. The MADRS-S ninth item scores from 0 to 6, with a higher score indicating higher severity of suicidal ideation.

After the 4 weeks of access to the intervention, participants completed two standardised self-assessed questionnaires measuring treatment credibility and usability. A five-item version of Credibility/Expectancy Questionnaire (CEQ)^34^ was used for measuring treatment credibility, and the System Usability Scale (SUS)^35^ was used for measuring usability. The CEQ ranges from 0 to 10 points per item and 0 to 50 points in total, where a higher score reflects better treatment credibility. The SUS consists of ten items, with a range of 0–4 points per item. The items are summed up and calculated with a formula resulting in a total score between 0 and 100, with a higher score reflecting better system usability. Recent research suggests that individual items of the SUS can be a valuable addition to the total score.^36^

Measures on behavioural engagement with the intervention were retrieved from the treatment platform after the 4 weeks of access to the intervention. The following measures were retrieved: the number of logins to the platform, the number of participants who used the intervention at least once, the number of problem-solving attempts initiated, the total number of generated solutions, the mean number of generated solutions per initiated problem-solving attempt and the number of participants who completed at least one evaluation of a problem-solving attempt.

Before treatment started, participants again completed the PHQ-9,^29^ GAD-7^30^ and the ninth item of the MADRS-S,^31^ and did so at the end of every week during access to the intervention, including after the final week. While the ninth item on MADRS-S (suicidal ideation) was only assessed for health-monitoring reasons, the scores on the PHQ-9 and GAD-7 were used to assess treatment improvement. During the end of the second and fourth week, participants completed the standardised and self-assessed 20-item version of the Negative Effects Questionnaire (NEQ),^37^ measuring negative effects of psychological interventions. The total score of the NEQ ranges from 0 to 80 points, and a higher score reflects more negative effects.

After using the intervention, participants completed a study-specific questionnaire concerning perceived user experience. This questionnaire comprised four questions focusing on whether the intervention was perceived as likable, whether the intervention was easy to understand, whether examples given felt relevant and whether functionality and information contributed to the participant feeling overwhelmed. All questions were answered on a four-point scale ranging from 0 (strongly disagree) to 3 (strongly agree). It was optional to add a free-text comment to the responses, as well as suggestions of overall improvements.

Finally, participants were contacted by telephone after the intervention access period, for a semi-structured interview concerning the experience of the intervention and format.

### Data analysis

Descriptive data were reported for all variables. The scores on the SUS were compared with a cut-off score of 50.9, reflecting the usability being perceived as ‘okay’, and a cut-off score of 71.4, reflecting the usability being perceived as ‘good’.^35^ Clinical improvement was measured by at least 20% symptom reduction on the PHQ-9 or GAD-7.^38^ Recovery was defined as at least 50% symptom reduction on the same measures.^39^ Since there are no current cut-off scores or consensus on how to interpret scores from the NEQ, descriptive statistics were used. Illustrative free-text comments from the study-specific questionnaire, as well as illustrative recurring comments from the interviews, were selected by the authors A.H. and M.K.

Participants who did not complete the SUS, CEQ and study-specific questionnaire could not be included in the summary or analyses of that data. For the PHQ-9 and GAD-7, participants who did not complete the post-treatment assessment were included in the analyses of the number of participants who reached a 20% and 50% symptom improvement, respectively. Missing data were, in those cases, conservatively handled as no symptom improvement. In the remaining results of the PHQ-9 and GAD-7, all available data were used. Behavioural engagement data were available for all participants, and thus no participant was excluded from the summary of those data.

## Results

### Demographics

The majority of participants were in a relationship and experienced both symptoms of depression and anxiety. See Table 1 for complete sample characteristics. Additionally, during the assessment, four out of 12 reported that they had attention-deficit hyperactivity disorder.

**Table 1:** Baseline characteristics of participants

Variable	Total (*N* = 12)
Female gender, *n* (%)	6 (50%)
Age, mean (s.d.) [range]	34.3 (11.1) [22−54]
In a relationship, *n* (%)	9 (75%)
Occupational status, *n* (%)	
Employed full time	4 (33%)
Employed part-time	5 (42%)
Student	2 (17%)
Unemployed	1 (8%)
Education, *n* (%)	
Primary school	1 (8%)
Secondary school	4 (33%)
University	7 (59%)
PHQ-9 screening ≥5, *n* (%)	12 (100%)
PHQ-9 screening, mean (s.d.) [range]	13.9 (4.5) [6−20]
GAD-7 screening ≥5, *n* (%)	11 (92%)
GAD-7 screening, mean (s.d.) [range]	11.6 (5.6) [4−21]
Ninth item of MADRS-S screening, mean (s.d.) [range]	1.9 (0.7) [1−3]

PHQ-9, Patient Health Questionnaire-9; GAD-7, Generalized Anxiety Disorder-7; MADRS-S, Montgomery–Åsberg Depression Rating Scale – Self Assessment.

### Missing data

The number of participants who had missing values at post-treatment assessment on the CEQ, SUS and the study-specific questionnaire was two (17%). Three (25%) participants had missing values on the PHQ-9 and GAD-7 after the first week of the intervention, five (42%) participants had missing values after the second week of the intervention, four (33%) participants had missing values after the third week of the intervention and two (17%) participants had missing values after the fourth week of the intervention (i.e. post-treatment assessment). At the midpoint assessment of the NEQ, four individuals (33%) had missing values, and two individuals (17%) had missing values at the post-treatment assessment of NEQ. Only one person did not complete the telephone-based interview post-treatment. Behavioural engagement data were available for all participants.

### Treatment credibility and usability

**Fig. 3:**
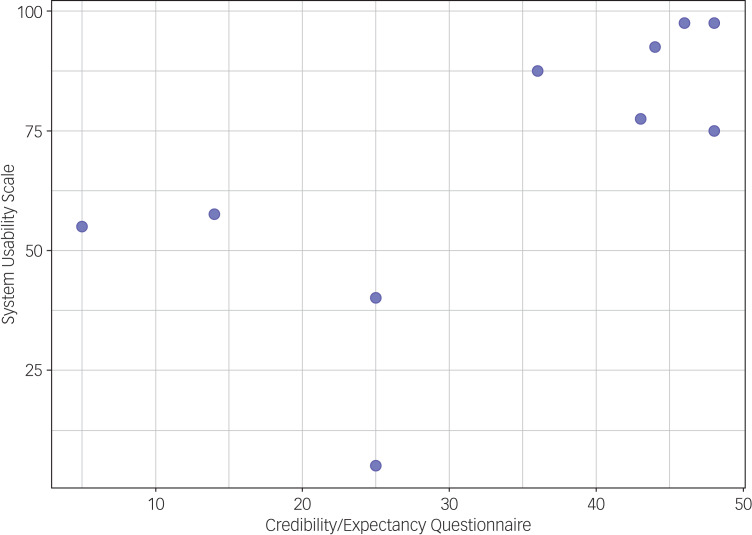
Scores on the Credibility/Expectancy Questionnaire and System Usability Scale for each individual.

The mean for the sum of the CEQ among those who completed the post-treatment assessment (*n* = 10) was 33.4 (s.d. 15.3, range 5–48, median 39.5). See Figure 3 for scores on the CEQ and SUS for each individual, and Table 2 for outcomes on each item of the CEQ.

**Table 2:** Outcomes on items of the Credibility/Expectancy Questionnaire, ranging from 0–10 points, with a higher score indicating a better treatment credibility (*N* = 10)

Item	Median	Mean	s.d.
How logical does this kind of intervention seem to you?	8.0	7.3	2.8
How successful do you think this kind of intervention will be in reducing your symptoms?	8.5	6.6	3.9
How confident would you be in recommending this intervention to a friend who experiences similar problems?	9.0	6.5	4.3
How successful do you think this kind of intervention would be for other similar problems?	10.0	7.8	3.4
How much improvement in your symptoms do you think will occur after having engaged in this intervention?	6.5	5.2	3.4

Among those who completed the post-treatment assessment (*n* = 10), the mean for the sum of the SUS was 68.5 (s.d. 29.6, range 5–97.5, median 76.3). One individual was an extreme outlier, with a score of five. Without this individual (*n* = 9), the mean for the sum of the SUS was 75.6 (s.d. 20.6, range 40–97.5, median 77.5). See Table 3 for outcomes on each item of the SUS. A total of eight out of ten individuals (80%) scored equal to or above the cut-off value indicating ‘okay’ usability. A total of six out of ten individuals (60%) scored equal to or above the cut-off value indicating ‘good’ usability.

**Table 3:** Outcomes on items of the System Usability Scale, ranging from 0 to 4 points, with a higher score indicating a better system usability (*N* = 10)

Item	Median	Mean	s.d.
I think that I would like to use this system frequently	3.0	2.4	1.8
I found the system unnecessarily complex^a^	3.0	2.7	1.3
I thought the system was easy to use	2.5	2.3	1.3
I think that I would need the support of a technical person to be able to use this system^a^	4.0	3.5	1.3
I found the various functions in this system were well integrated	3.0	2.6	1.1
I thought there was too much inconsistency in this system^a^	4.0	3.0	1.3
I would imagine that most people would learn to use this system very quickly	3.0	2.8	1.4
I found the system very cumbersome to use^a^	2.5	2.1	1.5
I felt very confident using the system	4.0	3.3	1.3
I needed to learn a lot of things before I could get going with this system^a^	3.5	2.7	1.6

a. Reversed item, with a higher score indicating better system usability.

### Behavioural engagement

A total of nine out of 12 individuals used the intervention at least once. Out of these nine participants, five individuals completed at least one evaluation of a problem-solving attempt. See Table 4 for outcomes on the behavioural engagement measures.

**Table 4:** Outcomes on behavioural engagement measures (*N* = 12)

Variable	Median	Mean	s.d.
Number of logins to the platform	7	11.4	10.5
Number of problem-solving attempts initiated	2	2.8	2.9
Total number of generated solutions	8	10.1	8.0
Mean number of generated solutions per initiated problem-solving attempt	3	2.7	1.2

### Symptoms of depression and anxiety

The participants with post-treatment assessment data (*n* = 10) showed a median symptom improvement on the PHQ-9 from screening to post-treatment of 2 points (16% improvement, interquartile range (IQR) = −0.5 to 4.8). The corresponding median symptom improvement on the GAD-7 was 3.5 points (22% improvement, IQR = −0.8 to 4.0).

**Fig. 4:**
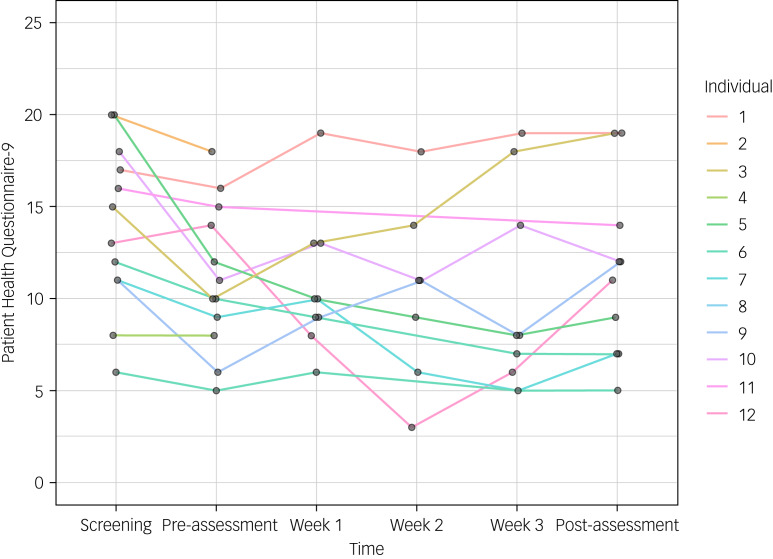
Individual change in scores on the Patient Health Questionnaire-9 at all measurement points.

**Fig. 5:**
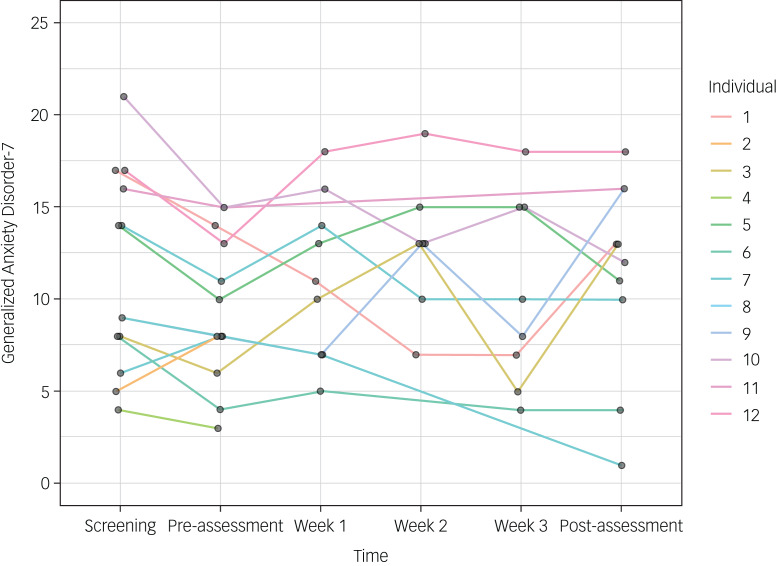
Individual change in scores on the Generalized Anxiety Disorder-7 at all measurement points.

From pre-treatment to post-treatment, participants who completed the post-assessment (*n* = 10) showed a median symptom improvement on the PHQ-9 of 0.5 points (3% improvement, IQR = −2.5 to 2.8). On the GAD-7, participants showed a median symptom deterioration from pre-treatment to post-treatment of 0.5 points (3% deterioration, IQR = −4.0 to 1.0). See Table 5 for the number of participants who reached at least 20% and 50% symptom improvement on the PHQ-9 and GAD-7, respectively, and Figures 4 and 5 for individual change in scores on these scales.

**Table 5:** Number of participants who reached a 20% and 50% symptom improvement on the Patient Health Questionnaire-9 and Generalized Anxiety Disorder-7, respectively, from screening to post-treatment and pre- to post-treatment

Instrument	Symptom improvement of at least	Period of change	Total (*N* = 12), *n* (%)
PHQ-9	20%	Screening to post-treatment	4 (33%)
		Pre- to post-treatment	4 (33%)
	50%	Screening to post-treatment	1 (8%)
		Pre- to post-treatment	0 (0%)
GAD-7	20%	Screening to post-treatment	6 (50%)
		Pre- to post-treatment	2 (17%)
	50%	Screening to post-treatment	2 (17%)
		Pre- to post-treatment	1 (8%)

PHQ-9, Patient Health Questionnaire-9; GAD-7, Generalized Anxiety Disorder-7.

### Negative effects

The mean score on the NEQ at the mid-assessment (*n* = 8) was 4.9 points (s.d. 1.9, range 3–9, median 4 points). At the post-assessment (*n* = 10), the mean score was 6.3 points (s.d. 4.9, range 3–17, median 4 points).

No serious negative effects were reported. Among the free-text reports on the NEQ, it was pointed out by a few participants that not being able to spend as much time as intended with the intervention generated a certain amount of guilt or stress.

### Experience with the intervention

See Table 6 for outcomes on the study-specific questionnaire.

**Table 6:** Outcomes on the study-specific questionnaire ranging from 0 (strongly disagree) to 3 (strongly agree) points (*N* = 10)

Variable	Median	Mean	s.d.
Likable	2.0	1.4	1.1
Easy to understand	2.0	2.1	0.7
Relevant examples	2.0	1.8	0.8
Overwhelmed	2.0	1.9	1.2

Participants reported positive and negative aspects of the intervention, as well as suggestions of improvements, in the free-text section. See Table 7 for selected illustrative quotes.

**Table 7:** Illustrative quotes from the free-text section in the study-specific questionnaire

Item		Quotes
Likable	Positive aspect	‘It makes it feel like I have started to unravel my problems and come closer to a solution.’
	Negative aspect	‘I experienced extreme anxiety by only starting to write down my problems.’
Easy to understand	Positive aspect	‘Good structure and good examples that make it easy to understand what to expect.’
	Negative aspect	‘I am not fully certain about how and what to write in each step. This makes it difficult for me, but it probably has more to do with me than the intervention.’
Relevant examples	Positive aspect	‘Most of the examples are highly relatable.’
	Negative aspect	‘The examples are definitely relevant, but I would like to see more examples of what to do if something is out of one's control.’
Overwhelmed	Positive aspect	‘The information is well-distributed across each part of the intervention, and to only gain access to one step at a time makes it feel less overwhelming. One is automatically forced to do one thing at a time, since anything else is simply not possible.’
	Negative aspect	‘I panicked about the stepwise access of the different parts of the content and not being able to look at forthcoming steps before I got there.’
Suggestions of possible improvements		‘Push notifications or daily reminders concerning one's ongoing problem-solving attempts.’ ‘An overview of all the solutions I have chosen to try.’

The average time for each interview was 10 min. In the interviews, most participants reported that they found the intervention helpful and that they appreciated the possibility to divide problems and solutions into manageable chunks. However, many stated that they did not use the intervention as much as they would have wanted. Some participants could not give any meaningful report because of insufficient usage of the intervention. A few participants said that they had expected that the intervention would generate solutions to their unique problems. However, some of these participants did state that after they had started to use the intervention, they were pleased with being prompted to reflect on their problems by themselves. One suggestion was to add more examples of problems and solutions.

One participant reported that although the intervention was easy to use, they had problems with motivation and keeping focus, and did not find the intervention helpful for staying concentrated and remembering to complete tasks. Among those who experienced difficulties remembering to use the intervention, one suggestion was to implement a function making it possible to choose intervals for getting reminders concerning remembering to work with the intervention.

## Discussion

This study aimed to evaluate the feasibility of a self-guided and monitored digital problem-solving intervention for patients awaiting treatment for depression or anxiety in routine psychiatric care. The patients were recruited from a Swedish routine psychiatric clinic, and most participants experienced symptoms of both depression and anxiety.

A majority of the participants found the intervention to be both credible and usable. A total of six out of ten individuals scored equal to or above the treatment credibility mean score of 33.03 points (s.d. 8.47) reported in internet-based CBT for depression in Swedish routine psychiatric care.^40^ Although participants found the intervention to be credible overall, they were not convinced that they would reach a high symptom improvement after having engaged with the intervention. One explanation to this could be the framing of the intervention as an intervention for the phase in-between assessment and treatment. It is unclear whether it would have made any difference to participants’ expectations of symptom improvement if the intervention would have been introduced as a possible replacement to the treatment they were awaiting.

A majority of the participants rated the usability above the threshold for ‘good’. When the extreme outlier was withdrawn from the analysis, the average usability score corresponded with this, with a value of 75.6 out of 100. This value is comparable to the total mean score of 67.85 points (s.d. 16.28) on the SUS found in a study among mental health professionals rating the usability of internet-based CBT for depression.^41^ The result is also comparable to the ratings of participants with clinical depression in a study investigating the usability of a self-guided mobile app-based intervention for depression, where the total mean score on the SUS was 86.00 (s.d. 10.84).^42^ It should, however, be noted that the sample of end-users in that study consisted of a total of five participants, who were paid after they completed the trial.

Among the participants in the current study, a few items on the SUS stood out as especially positive. For example, participants did not, on average, feel like they were in the need of technical support to be able to use the intervention and its system. Furthermore, the participants felt very confident using the system, and they did not find too much inconstancy in the system. This is encouraging, since a psychiatric population may experience cognitive difficulties affecting the daily life, such as focusing on and processing information.^43^ The scoring indicating low need of technical support can be interpreted as the intervention being delivered in a way that is easy enough to process in a self-guided format for a psychiatric population.

With an average of around 11 logins to the treatment platform and three problem-solving attempts, the engagement level can be considered encouraging compared with other self-guided interventions. As a contrast, there was a 7-week self-guided intervention in Sweden that targeted mental health problems, where the average number of logins was six and participants on average completed 0.2 treatment modules.^44^ This intervention did not, however, include clinical monitoring of symptoms, which may have driven the higher behavioural engagement in our study. Another relevant comparison would be the online therapist-guided problem-solving intervention that was evaluated for patients on the waiting list for routine mental healthcare in The Netherlands, by Kenter et al.^15^ That study reported higher engagement level than we observed in our study, with two-thirds of participants completing three or more problem-solving lessons. Notably, therapists in the intervention studied by Kenter et al spent approximately 40 minutes per lesson on feedback (a majority of patients completed three or more lessons), whereas the intervention in our study was completely self-guided.

Symptoms of depression and anxiety were overall stable among the sample during the intervention period. Only a few participants reached the threshold for clinical improvement on symptoms of depression or anxiety. Some individuals did reach some symptom improvement, but for most participants, this intervention was not a substitute for the regular treatment they were waiting for. Furthermore, some individuals deteriorated slightly on depression or anxiety symptom scores, but we did not find any evidence of this being a result of negative effects from the intervention. However, these clinical effects should be regarded as preliminary because of the small sample size and uncontrolled study design. Should a future randomised clinical trial show a similar trend in treatment effects of this intervention, it would indicate that the value of using this kind of intervention as a part of a stepped-care model might be limited.^45^ Taking into account the sufficient credibility and usability ratings of the intervention, and relatively high behavioural engagement, the intervention could still be of value as a meaningful activity for patients awaiting treatment for their psychiatric conditions, potentially decreasing the risk of prolonged or increased suffering. In its current state, the problem-solving intervention should be considered as an addition to, rather than replacement for, other psychiatric services.

Finally, suggestions of possible improvements of the intervention from the patients included more flexible options of reminders to remember to work with one's problems and an improved overview of the solutions chosen to work with. In an improved version of the problem-solving intervention, these suggestions should be considered to be implemented.

This study investigated the feasibility of providing a self-guided and monitored digital problem-solving intervention for patients awaiting treatment for depression or anxiety in routine psychiatric care. Participants found the intervention both credible and usable, and the engagement with the intervention was comparable to other self-guided interventions deemed sufficient. In summary, a problem-solving intervention in a self-guided and monitored format may be a beneficial intervention for patients in-between the phase of assessment and access to psychiatric treatment within regular healthcare.

### Limitations

This study had several limitations that need to be acknowledged. First, this study was a feasibility study, uncontrolled and consisted of a small sample size. Therefore, all effect estimates must be considered very preliminary. Second, since the aim of this study was to conduct an initial evaluation of whether the intervention was feasible in the current context, the period of usage of the intervention was chosen to be limited to 4 weeks. In an improved trial of the potential benefit of the intervention as a part of a stepped-care model or help during the waiting period for treatment, the intervention could be chosen to be provided during the complete waiting period, whether that would be over weeks or months. Third, a significant number of participants assessed for eligibility were excluded because of severe suicidal ideation, leaving the results deprived of input from patients with such problems. Finally, a majority of the participants were university educated, making the data representative for similar populations.

### Clinical implications and future research

The results from this study indicate that a self-guided and monitored digital problem-solving intervention could be a feasible intervention to provide for patients in routine care while they are awaiting psychiatric treatment. However, future studies concerning treatment effects are needed.

The effects of the intervention on symptoms of depression and anxiety need to be evaluated. Preferably, the intervention could be evaluated when provided to patients during the complete waiting period in-between assessment and access to treatment, rather than during a specific number of weeks.

## Data Availability

Deidentified participant data will be made available upon reasonable request to the corresponding author, A.H.

## References

[1] Richter D, Wall A, Bruen A, Whittington R. Is the global prevalence rate of adult mental illness increasing? Systematic review and meta-analysis. Acta Psychiatr Scand 2019; 140(5): 393–407. 31393996 10.1111/acps.13083

[2] Layard R. *The Economics of Mental Health*. IZA World of Labor, 2017 (http://wol.iza.org/articles/economics-of-mental-health).

[3] König H, König H-H, Konnopka A. The excess costs of depression: a systematic review and meta-analysis. Epidemiol Psychiatr Sci 2020; 29: e30. 10.1017/S2045796019000180 PMC8061284 30947759

[4] Carlbring P, Andersson G, Cuijpers P, Riper H, Hedman-Lagerlöf E. Internet-based vs. face-to-face cognitive behavior therapy for psychiatric and somatic disorders: an updated systematic review and meta-analysis. Cogn Behav Ther 2018; 47(1): 1–18. 29215315 10.1080/16506073.2017.1401115

[5] Patel V, Maj M, Flisher AJ, De Silva MJ, Koschorke M, Prince M, Reducing the treatment gap for mental disorders: a WPA survey. World Psychiatry 2010; 9(3): 169–76. 20975864 10.1002/j.2051-5545.2010.tb00305.x PMC2953637

[6] Furukawa TA, Noma H, Caldwell DM, Honyashiki M, Shinohara K, Imai H, Waiting list may be a nocebo condition in psychotherapy trials: a contribution from network meta-analysis. Acta Psychiatr Scand 2014; 130(3): 181–92. 24697518 10.1111/acps.12275

[7] Scogin FR, Hanson A, Welsh D. Self-administered treatment in stepped-care models of depression treatment. J Clin Psychol 2003; 59(3): 341–9. 12579549 10.1002/jclp.10133

[8] van Straten A, Cuijpers P, Smits N. Effectiveness of a web-based self-help intervention for symptoms of depression, anxiety, and stress: randomized controlled trial. J Med Internet Res 2008; 10(1): e7. 18364344 10.2196/jmir.954 PMC2483843

[9] Kleiboer A, Donker T, Seekles W, van Straten A, Riper H, Cuijpers P. A randomized controlled trial on the role of support in internet-based problem solving therapy for depression and anxiety. Behav Res Ther 2015; 72: 63–71. 26188373 10.1016/j.brat.2015.06.013

[10] Warmerdam L, van Straten A, Twisk J, Riper H, Cuijpers P. Internet-based treatment for adults with depressive symptoms: randomized controlled trial. J Med Internet Res 2008; 10(4): e44. 19033149 10.2196/jmir.1094 PMC2629364

[11] Zhang A, Park S, Sullivan JE, Jing S. The effectiveness of problem-solving therapy for primary care patients’ depressive and/or anxiety disorders: a systematic review and meta-analysis. J Am Board Fam Med 2018; 31(1): 139–50. 29330248 10.3122/jabfm.2018.01.170270

[12] Cuijpers P, de Wit L, Kleiboer A, Karyotaki E, Ebert DD. Problem-solving therapy for adult depression: an updated meta-analysis. European Psychiatry 2018; 48(1): 27–37. 29331596 10.1016/j.eurpsy.2017.11.006

[13] Titov N, Dear BF, Johnston L, Lorian C, Zou J, Wootton B, Improving adherence and clinical outcomes in self-guided internet treatment for anxiety and depression: randomised controlled trial. PLoS One 2013; 8(7): e62873. 23843932 10.1371/journal.pone.0062873 PMC3701078

[14] Titov N, Dear BF, Staples LG, Terides MD, Karin E, Sheehan J, Disorder-specific versus transdiagnostic and clinician-guided versus self-guided treatment for major depressive disorder and comorbid anxiety disorders: a randomized controlled trial. J Anxiety Disord 2015; 35: 88–102. 26422822 10.1016/j.janxdis.2015.08.002

[15] Kenter R, Warmerdam L, Brouwer-Dudokdewit C, Cuijpers P, van Straten A. Guided online treatment in routine mental health care: an observational study on uptake, drop-out and effects. BMC Psychiatry 2013; 13: 43. 23368894 10.1186/1471-244X-13-43 PMC3577663

[16] Reins JA, Ebert DD, Lehr D, Riper H, Cuijpers P, Berking M. Internet-based treatment of major depression for patients on a waiting list for inpatient psychotherapy: protocol for a multi-centre randomised controlled trial. BMC Psychiatry 2013; 13: 318. 24279841 10.1186/1471-244X-13-318 PMC4222859

[17] Naeem F, Munshi T, Gratzer D, Rodie D, Irfan M, Rao S, Video intervention for the psychiatric waiting room: proof-of-concept randomised controlled trial of RESOLVE (Relaxation Exercise SOLVing problem and cognitive Errors). BJPsych Open 2019; 5(5): e77. 31488227 10.1192/bjo.2019.59 PMC6737517

[18] Neben T, Seeger A-M, Kramer T, Knigge S, White AJ, Alpers GW. Make the most of waiting: theory-driven design of a pre-psychotherapy mobile health application. *Twenty-Second Americas Conference on Information Systems (San Diego, USA, 11–14 Aug 2016).* Association for Information Systems, 2016.

[19] Beatty L, Binnion C. A systematic review of predictors of, and reasons for adherence to online psychological interventions. IntJ Behav Med 2016; 23(6): 776–94. 26957109 10.1007/s12529-016-9556-9

[20] Ossebaard HC, Seydel ER, van Gemert-Pijnen L. Online usability and patients with long-term conditions: a mixed-methods approach. Int J Med Inform 2012; 81(6): 374–87. 22261086 10.1016/j.ijmedinf.2011.12.010

[21] Alfonsson S, Olsson E, Hursti T. Motivation and treatment credibility predicts dropout, treatment adherence, and clinical outcomes in an internet-based cognitive behavioral relaxation program: a randomized controlled trial. J Med Internet Res 2016; 18(3): e52. 26957354 10.2196/jmir.5352 PMC4804106

[22] El Alaoui S, Ljótsson B, Hedman E, Kaldo V, Andersson E, Rück C, Predictors of symptomatic change and adherence in internet-based cognitive behaviour therapy for social anxiety disorder in routine psychiatric care. PLoS One 2015; 10(4): e0124258. 25893687 10.1371/journal.pone.0124258 PMC4404057

[23] Davis FD. Perceived usefulness, perceived ease of use, and user acceptance of information technology. MIS Q 1989; 13(3): 319.

[24] Eysenbach G. The law of attrition. J Med Internet Res 2005; 7(1): e11. 15829473 10.2196/jmir.7.1.e11 PMC1550631

[25] Torous J, Nicholas J, Larsen ME, Firth J, Christensen H. Clinical review of user engagement with mental health smartphone apps: evidence, theory and improvements. Evid Based Ment Health 2018; 21(3): 116–9. 29871870 10.1136/eb-2018-102891 PMC10270395

[26] Perski O, Blandford A, West R, Michie S. Conceptualising engagement with digital behaviour change interventions: a systematic review using principles from critical interpretive synthesis. Behav Med Pract Policy Res 2017; 7(2): 254–67. 10.1007/s13142-016-0453-1 PMC5526809 27966189

[27] Alberts NM, Law EF, Chen AT, Ritterband LM, Palermo TM. Treatment engagement in an internet-delivered cognitive behavioral program for pediatric chronic pain. Internet Interv 2018; 13: 67–72. 30206521 10.1016/j.invent.2018.07.005 PMC6112105

[28] Abbott JH. The distinction between randomized clinical trials (RCTs) and preliminary feasibility and pilot studies: what they are and are not. J Orthop Sports Phys Ther 2014; 44(8): 555–8. 25082389 10.2519/jospt.2014.0110

[29] Kroenke K, Spitzer RL, Williams JBW. The PHQ-9: validity of a brief depression severity measure. J Gen Intern Med 2001; 16(9): 606–13. 11556941 10.1046/j.1525-1497.2001.016009606.x PMC1495268

[30] Spitzer RL, Kroenke K, Williams JBW, Löwe B. A brief measure for assessing generalized anxiety disorder: the GAD-7. Arch Intern Med 2006; 166(10): 1092. 16717171 10.1001/archinte.166.10.1092

[31] Svanborg P, Åsberg M. A new self-rating scale for depression and anxiety states based on the Comprehensive Psychopathological Rating Scale. Acta Psychiatr Scand 1994; 89(1): 21–8. 8140903 10.1111/j.1600-0447.1994.tb01480.x

[32] Hedman E, Ljótsson B, Kaldo V, Hesser H, El Alaoui S, Kraepelien M, Effectiveness of internet-based cognitive behaviour therapy for depression in routine psychiatric care. J Affect Disord 2014; 155: 49–58. 24238951 10.1016/j.jad.2013.10.023

[33] Parasuraman S, Sam AT, Yee SWK, Chuon BLC, Ren LY. Smartphone usage and increased risk of mobile phone addiction: a concurrent study. Int J Pharm Invest 2017; 7(3): 7. 10.4103/jphi.JPHI_56_17 PMC5680647 29184824

[34] Borkovec TD, Nau SD. Credibility of analogue therapy rationales. J Behav Ther Exp Psychiatry 1972; 3(4): 257–60.

[35] Lewis JR. The System Usability Scale: past, present, and future. Int J Hum Comput Interact 2018; 34(7): 577–90.

[36] Lewis JR, Sauro J. Item benchmarks for the system usability scale. J Usability Stud 2018; 13(3): 158–67.

[37] Rozental A, Kottorp A, Forsström D, Månsson K, Boettcher J, Andersson G, The Negative Effects Questionnaire: psychometric properties of an instrument for assessing negative effects in psychological treatments. Behav Cogn Psychother 2019; 47(5): 559–72. 30871650 10.1017/S1352465819000018

[38] Kounali D, Button KS, Lewis G, Gilbody S, Kessler D, Araya R, How much change is enough? Evidence from a longitudinal study on depression in UK primary care. Psychol Med [Epub ahead of print] 3 Nov 2020. Available from: 10.1017/S0033291720003700. PMC9340848 33138872

[39] Richards DA, Suckling R. Improving access to psychological therapies: phase IV prospective cohort study. Br J Clin Psychol 2009; 48(4): 377–96. 19208291 10.1348/014466509X405178

[40] El Alaoui S, Ljótsson B, Hedman E, Svanborg C, Kaldo V, Lindefors N. Predicting outcome in internet-based cognitive behaviour therapy for major depression: a large cohort study of adult patients in routine psychiatric care. PLoS ONE 2016; 11(9): e0161191. 27618548 10.1371/journal.pone.0161191 PMC5019371

[41] Mol M, van Schaik A, Dozeman E, Ruwaard J, Vis C, Ebert DD, Dimensionality of the System Usability Scale among professionals using internet-based interventions for depression: a confirmatory factor analysis. BMC Psychiatry 2020; 20: 218. 32398111 10.1186/s12888-020-02627-8 PMC7216472

[42] Fuller-Tyszkiewicz M, Richardson B, Klein B, Skouteris H, Christensen H, Austin D, A mobile app–based intervention for depression: end-user and expert usability testing study. JMIR Ment Health 2018; 5(3): e54. 30139722 10.2196/mental.9445 PMC6127496

[43] Knight MJ, Baune BT. Cognitive dysfunction in major depressive disorder. Curr Opin Psychiatry 2018; 31(1): 26–31. 29076892 10.1097/YCO.0000000000000378

[44] Nilsson A, Sörman K, Klingvall J, Ovelius E, Lundberg J, Hellner C. MyCompass in a Swedish context – lessons learned from the transfer of a self-guided intervention targeting mental health problems. BMC Psychiatry 2019; 19: 51. 30704424 10.1186/s12888-019-2039-1 PMC6357356

[45] Bower P, Gilbody S. Stepped care in psychological therapies: access, effectiveness and efficiency: narrative literature review. Br J Psychiatry 2005; 186(1): 11–7. 15630118 10.1192/bjp.186.1.11

