# A self-guided and monitored digital problem-solving intervention for patients with symptoms of depression or anxiety on the waiting list for treatment in routine psychiatric care: feasibility study – CORRIGENDUM

**DOI:** 10.1192/bjo.2022.72

**Published:** 2022-06-20

**Authors:** Amira Hentati, Erik Forsell, Brjánn Ljótsson, Nils Lindefors, Martin Kraepelien

**Keywords:** Anxiety disorders, cognitive–behavioural therapies, depressive disorders, digital intervention, routine psychiatry

The authors would like to make two corrections to the text:

**Methods**

All questions were answered on a four-point scale ranging from 0 (strongly disagree) to 3 (strongly agree). **The scoring of the fourth item was reversed, and thus a higher score signified feeling less overwhelmed.**

**Table 6**

Outcomes on the study-specific questionnaire ranging from 0 (strongly disagree) to 3 (strongly agree) points, **with reversed scoring of the fourth item** (*N* = 10)

The authors sincerely apologise for the errors.
